# Effects of *Wolbachia* on ovarian apoptosis in *Culex quinquefasciatus* (Say, 1823) during the previtellogenic and vitellogenic periods

**DOI:** 10.1186/s13071-017-2332-0

**Published:** 2017-08-25

**Authors:** Fabio Almeida, Lincoln Suesdek

**Affiliations:** 1 Laboratório de Parasitologia, Instituto Butantan, São Paulo, Brazil; 2 Instituto de Medicina Tropical, Universidade de São Paulo, São Paulo, Brazil

**Keywords:** Mosquito, Cell death, Reproduction, Atresia, Oogenesis

## Abstract

**Background:**

Apoptosis is programmed cell death that ordinarily occurs in ovarian follicular cells in various organisms. In the best-studied holometabolous insect, *Drosophila*, this kind of cell death occurs in all three cell types found in the follicles, sometimes leading to follicular atresia and egg degeneration. On the other hand, egg development, quantity and viability in the mosquito *Culex quinquefasciatus* are disturbed by the infection with the endosymbiont *Wolbachia*. Considering that *Wolbachia* alters reproductive traits, we hypothesised that such infection would also alter the apoptosis in the ovarian cells of this mosquito. The goal of this study was to comparatively describe the occurrence of apoptosis in *Wolbachia*-infected and uninfected ovaries of *Cx. quinquefasciatus* during oogenesis and vitellogenesis. For this, we recorded under confocal microscopy the occurrence of apoptosis in all three cell types of the ovarian follicle. In the first five days of adult life we observed oogenesis and, after a blood meal, the initiation step of vitellogenesis.

**Results:**

Apoptoses in follicular cells were found at all observation times during both oogenesis and vitellogenesis, and less commonly in nurse cells and the oocyte, as well as in atretic follicles. Our results suggested that apoptosis in follicular cells occurred in greater numbers in infected mosquitoes than in uninfected ones during the second and third days of adult life and at the initiation step of vitellogenesis.

**Conclusions:**

The presence of *Wolbachia* leads to an increase of apoptosis occurrence in the ovaries of *Cx. quinquefasciatus*. Future studies should investigate if this augmented apoptosis frequency is the cause of the reduction in the number of eggs laid by *Wolbachia*-infected females. Follicular atresia is first reported in the previtellogenic period of oogenesis. Our findings may have implications for the use of *Wolbachia* as a mosquito and pathogens control strategy.

**Electronic supplementary material:**

The online version of this article (doi:10.1186/s13071-017-2332-0) contains supplementary material, which is available to authorized users.

## Background

*Culex quinquefasciatus* is a common house mosquito, and because they pierce the hosts’ skin to consume blood, they are a competent vector of neurotropic viruses, such as West Nile Virus and human and veterinary encephalitis viruses. They can also transmit filarial worms and cause substantial nocturnal discomfort and allergic reactions [1]. Despite its medical and veterinary importance, few factors regarding reproduction and fitness of this insect have been explored, and one of these factors is the cell death of ovarian cells, that have an important role on the quantities of laid eggs [2, 3].

We believe that cell death of ovarian cells might be influenced by presence of *Wolbachia*. Approximately half of all insect species are infected by *Wolbachia* [4–6], and several Brazilian populations of *Culex quinquefasciatus* are naturally infected by these bacteria [7]. *Wolbachia* is maternally transmitted, and it induces incompatible crossings (cytoplasmic incompatibility) and reproductive disturbance in most of its arthropod hosts. Because of this, researchers have proposed the use of this bacterium as a new strategy to control insects and thereby the diseases carried by the mosquitoes (see [8]) and, so far, two main strategies can be used. The first is using cytoplasmic incompatibility for population substitution and control [9, 10], and the second is related to the influence of this bacterium on the replication of pathogens that infect the vector concomitantly, leading to non-transmission of the pathogen [8].

Apoptosis is genetically programmed cell death (PCD), which commonly occurs because of physiological necessity or because cells fail to develop [11]. It is advantageous compared to necrosis, because extravasation of cellular material does not occur, preventing the inflammatory response, and nutrients can be absorbed and utilised by the surrounding tissue [12]. Programmed cell death has been described in ovaries from different organisms [13, 14], including in the insect holometabolous model, *Drosophila* [2, 15, 16]. PCD in reproductive tissue of *Drosophila* is well described and is known to occur at different times of oogenesis and in different ways [17], but this kind of study is scarce in mosquitoes.

It is known that one of the factors that can induce cell death is the presence of the iron molecule, which causes oxidative stress in cells, leading to apoptosis [18]. In some mosquitoes, it was described that the presence of *Wolbachia* causes an increase of the transcripts related to the fight against oxidative stress [19, 20].

The occurrence of natural follicular death (atresia) has been described in some mosquitoes, such as *Aedes aegypti*, which seems to occur primarily between 26 and 30 h after a blood meal [3]. In *Culex pipiens pallens*, it occurs in the first stages of the vitellogenic period and between the second and third days after a blood meal [21–23]. In these studies, however, observations were made by indirect or non-specific methods, and information was not obtained for the previtellogenic period.

Mature ovaries of *Cx. quinquefasciatus* consists of ovarioles containing primary and secondary follicles [24]. Each primary follicle (which develops after a blood meal) has three types of cells. Follicular cells are responsible for transport of nutrients and the production of the chorion. Seven nurse cells are responsible for the synthesis of ribosomes and mRNA for the oocyte. Finally, the single oocyte is responsible for the accumulation of nutrients to be used by the embryo [25–27]. The secondary follicle is composed of undifferentiated cells and will turn into a primary follicle after oviposition [28].

The development of ovaries in adult mosquitoes is divided into two stages, the previtellogenic period (PVP; before the blood meal) and vitellogenic period (VP; after the blood meal). During the previtellogenic period, the action of the juvenile hormone triggers preparation of the ovarian follicle cells for absorption and processing of vitellogenin [29]. Next, the 20-hydroxyecdysone hormone inhibits the development of follicle cells, maintaining them in a resting stage until the blood meal [30]. The vitellogenic period begins with the blood meal, and the follicular cells begin the production of chorionic proteins. The fat body begins rapid production of vitellogenin and rapid intake through the oocyte by the vitellogenin receptor and by patency [31–33]. Patency is the development of intercellular channels between follicular cells that permit the transport of yolk from the hemolymph directly into the oocyte [34].

The time required for ovary development depends on the mosquito species. In *Cx. quinquefasciatus*, the previtellogenic phase is completed by approximately the fourth or fifth day of adult life and, the vitellogenic period is completed between the third and fourth day after a blood meal when the female is ready to lay eggs [32]. In previous work, we found that *Wolbachia* in *Cx. quinquefasciatus* caused cytoplasmic incompatibility, reduction in the number of eggs (in 4 consecutively gonotrophic cycles) and reduction of eggs viability (principally on the second gonotrophic cycle) [35].

Considering that the natural infection of *Wolbachia* significantly alters egg-related traits of *Cx. quinquefasciatus*, we hypothesised that this endosymbiont bacterium also alters the apoptosis in the ovarian cells. The aim of this study was to comparatively describe the occurrence of apoptosis in *Wolbachia*-infected and uninfected ovaries of *Cx. quinquefasciatus* during oogenesis, either in the previtellogenic and vitellogenic periods.

## Methods

### Animals

Founder specimens of *Cx. quinquefasciatus* were initially collected near the banks of the Pinheiros River, São Paulo City, Brazil (23°35′S, 46°41′W). The mosquitoes have been reared in a local insectary since 1995, and were raised at 26–28 °C and 70–80% relative humidity under a photoperiod of 12 h dark-12 h light. Larvae were fed with powdered fish food (Sera® Vipan, Heinsberg, Germany) and adults were fed a 10% sucrose solution ad libitum. When necessary, adult females were fed on BALB/c mice, anaesthetized with 80 mg kg^−1^ of ketamine and 10 mg kg^-1^ of xylazine hydrochloride. In 2005, the presence of *Wolbachia* was detected by electron microscopy and confirmed by PCR and sequencing [see 35].

### Detection of *Wolbachia*

Rapid molecular detection of the *wsp* gene was performed using *wsp* primers 183F and 691R, according to the methods of Zhou et al. [36]. Amplifications were checked by agarose electrophoresis. The offspring of 20 females that were PCR-positive for the presence of *Wolbachia* were selected to originate the *Wolbachia*-infected mosquito group (*w*Pip+).

### Tetracycline treatment to obtain uninfected mosquitoes (*w*Pip-)

Approximately 600 adult mosquitoes were fed with sucrose solution containing tetracycline hydrochloride solution (pH 7.0) at a final concentration of 1 mg ml^-1^ for seven days. Five days after treatment completion, female mosquitoes (parental generation) were allowed to feed on mice to initiate egg laying. Adults from the next generation (F1) were submitted to the same treatment and five days later were blood-fed (see details in [35]). After the individual oviposition, the treated F1 females were subjected to PCR, and the offspring of females (F2) who had no infection were separated. When they became adults, they were fed on mice, generating new offspring. After the fourth consecutive generation with no *Wolbachia* detection (20 females tested per generation), we began preparation of the infected (*w*Pip+) and uninfected (*w*Pip-) material for microscopy. From this point on, every two months PCRs were performed in both colonies to confirm *Wolbachia* status.

### Ovary morphology

Mosquito ovaries were dissected in 4% paraformaldehyde in phosphate buffered saline (1× PBS) at 12–24 h (1 day); 36–48 h (2 days); 60–72 h (3 days); 84–96 h (4 days); and 108–120 h (5 days) after adult emergence (PVP). On the sixth day of adult life, the mosquitoes were fed blood, and their ovaries were dissected at 6 h, 12 h, 24 h, 36 h, 48 h and 72 h after the blood meal (VP). Unlike the VP periods, the PVP periods were not precise because the emergence of mosquitoes did not occur synchronously; however, the mice were offered to mosquitoes for 1 h, after which they were removed from the cages.

The ovaries were maintained in the fixative solution for 30 to 60 min. Depending on the stage of development: 30 min for ovaries on the first and second day of PVP; 40 min for ovaries on 3–5 days of PVP and 6 to 12 h of VP; and 60 min for ovaries on 24 h or more of VP. The samples were processed using the terminal deoxynucleotidyl transferase dUTP nick marker end labelling (TUNEL) kit to detect apoptosis and atresia.

We used the TUNEL kit Click-It® Alexa Fluor® 488 Imaging Assay (Invitrogen, Carlsbad, USA) following the manufacturer’s instructions with modifications. The ovaries were permeabilized with Triton X-100 1% in PBS (1×) for 30–60 min (using the same pattern as the fixative), incubated with fluorescent nucleotides (fluorescein-12-dUTP) and the terminal enzyme deoxynucleotidyl transferase (TdT) for 60 min at 37 °C for the synthesis of the nucleotide tail. After 3 washes of 5 min each with 1× PBS/3% BSA (bovine serum albumin in phosphate buffered saline), the samples were transferred to nuclear marker TO-PRO®-3 647 stain for 30–60 min (using the same pattern as the fixative) at a the dilution of 1:100 in 1× PBS:3% BSA. Following fixation and labelling, the material was mounted on a slide with a coverslip, or between two coverslips, with antifade Vectashield Mounting Medium (Vector Labs, Burlingame, USA) and observed under confocal Laser Scanning Microscopy, LSM 510 META, Zeiss. The images with TUNEL marking were located with the LSM Image Browser program (Zeiss, Oberkochen, Germany).

### Statistical analyses

Twenty (20) ovaries from different females at each sampling period (first to fifth day during the PVP, and 6 h, 12 h and 24 h during the VP) per group (wPip+ and wPip-) were analysed to compare the number of apoptotic events. Statistical analyses were performed aiming to compare between infected and uninfected, separately at each time.

The parametrical Student’s *t*-test was used to compare between samples with normal distributions (Shapiro-Wilk), and the nonparametric Mann-Whitney test was used for those with non-normal and heteroscedastic distributions. Rejection level (alpha) was stipulated at 5%.

## Results

### Tetracycline treatment

The treatment with tetracycline was successful so that we were capable of originating an uninfected group. The disinfection was stable during the whole period of experiments, as confirmed by the regular PCR-checkings. After the treatment, no *Wolbachia*-positive individuals were detected (Additional file 1: Figure S1).

### General morphology of the ovaries

Using confocal microscopy, we described the developmental patterns of ovaries during several moments of the oogenesis and vitellogenesis. No ovarian morphological differences were observed between wPip+ and wPip- mosquitoes.

In the first day of the PVP it was not possible to identify follicles in the ovarian tissue; on the second day, follicles were noticeable, but it was not possible to distinguish between primary and secondary follicles (Additional file 2: Figure S2). On the other hand, between the third and fifth days of the PVP, primary and secondary follicles had distinguishable sizes, and ovarian development was apparently complete. Furthermore, at that point, the presence of nurse cells and/or oocyte in primary follicles was pronounced. At 24 h of the VP, the primary follicle exhibited an increased size, and the difference between nurse cells and the oocyte was already apparent, being the oocyte occupying most of the ovarian follicle (Additional file 3: Figure S3).

At 24 h of VP, it was necessary to dissect and separate the ovarioles from the ovary and assemble them between coverslips to permit laser penetration (of the confocal microscope) through the primary follicles. Even though, later stages of the VP could not be analysed because of chorion broad-spectrum autofluorescence and chorion thickness, which prevented laser penetration (Additional file 4: Figure S4). In general, despite the technical constraints, using the confocal approach we could identify the main developmental phases of mosquito eggs.

### Apoptosis of follicular cells

To quantify the apoptotic events in follicular cells and to compare it between infected and uninfected females, we observed under confocal microscopy, the ovaries labelled by TUNEL and TO-PRO in several occasions of the previtellogenic and vitellogenic periods. Using the Z-stack of the confocal microscope, only nuclei of cells that were simultaneously labelled with two fluorophores (TO-PRO and TUNEL) were regarded as apoptotic cells.

**Fig. 1:**
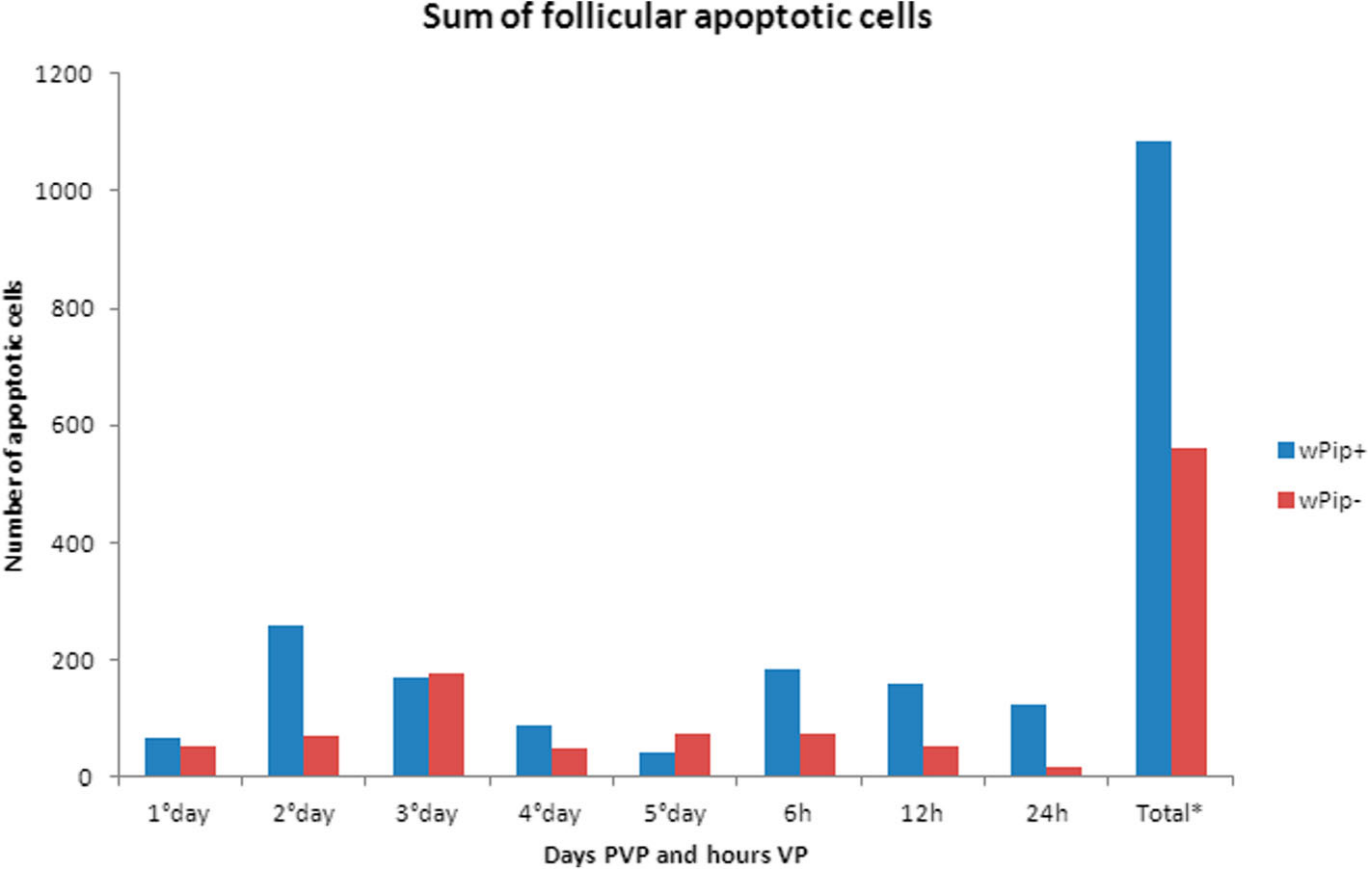
Total numbers of apoptotic follicular cells in PVP and VP. *Abbreviations*: PVP, previtellogenic period; VP, vitellogenic period; *w*Pip+, infected; *w*Pip-, uninfected

The sum of apoptotic events in ovary follicular cells of infected females was 1087 from all 160 analysed ovaries, whereas, in 160 ovaries from non-infected individuals, only 562 apoptotic events were detected. Regarding the total observations, the difference between *w*Pip+ and *w*Pip- was statistically significant (Mann-Whitney test, *U* = 9240, n_1_ = n_2_ = 160, *P* < 0.0001). The greatest contribution to this difference between both insect groups was on the second and third day of the PVP and 6 h and 12 h of the VP (Table 1; Fig. 1; Additional file 5: Table S1; Additional file 6: Figure S5). The difference between *w*Pip+ and *w*Pip- occurred mainly because of several apoptoses in secondary follicles (544 infected secondary infected follicles had at least one apoptotic cell, whereas it was 323 for uninfected secondary follicles, Mann-Whitney test, *U* = 5378.5, n_1_ = n_2_ = 120, *P* = 0.0007). Primary follicles contributed little to this difference as only 98 infected and 90 uninfected follicles had at least one apoptotic cell (Mann-Whitney test, *U* = 7182.5, n_1_ = n_2_ = 120, *P* = 0.9744). Inferential statistics quantitatively compared the presence of apoptotic follicular cells between infected and uninfected females at respective time points, during the previtellogenic and vitellogenic periods.

**Table 1:** Mean number (± standard error) of follicular apoptotic cells and comparative statistics between wPip+ and wPip- groups

Period of oogenesis		Mean no. of apoptotic follicular cells per ovary (1)	Mean no. of primary follicles that have at least 1 apoptotic event (2)	Mean no. of secondary follicles that have at least 1 apoptotic event (3)	Statistics^b^
PVP					
Day 1	*w*Pip+	3.3 ± 3.29	nd	nd	(1) *t* = 0.6285, *P* = 0.5334^c^
	*w*Pip-	2.55 ± 4.2	nd	nd	
Day 2^a^	*w*Pip+	12.85 ± 13.99**	nd	9.65 ± 5.83**	(1) *U* = 57.5, *P* < 0.0001; (3) *t* = 4.274, *P* < 0.0001
	*w*Pip-	3.45 ± 3.52**	nd	3.15 ± 3.5**	
Day 3	*w*Pip+	8.4 ± 5.25	0.4 ± 0.82**	7.55 ± 4.26	(1) *t* = 0.2246, *P* = 0.8081; (2) *U* = 81.5, *P* = 0.0013; (3) *t* = 0.9477, *P* = 0.3493
	*w*Pip-	8.8 ± 5.09	1.9 ± 1.8**	6.3 ± 4.08	
Day 4	*w*Pip+	4.43 ± 4.02	0.15 ± 0.37	3.6 ± 3.36	(1) *t* = 1.724, *P* = 0.0929; (2) *U* = 190, *P* = 0.7890; (3) *t* = 1.633, *P* = 0.1107
	*w*Pip-	2.5 ± 2.7	0.2 ± 0.41	2.1 ± 2.36	
Day 5	*w*Pip+	2.05 ± 2.26*	0.4 ± 0.82	1.35 ± 1.53*	(1) *t* = 2.106, *P* = 0.0418; (2) *U* = 145.5, *P* = 0.1382; (3) *t* = 2.088, *P* = 0.0435
	*w*Pip-	3.7 ± 2.68*	1 ± 1.34	2.55 ± 2.06*	
VP					
6 h	*w*Pip+	9.15 ± 3.67**	1.2 ± 1.58*	7.2 ± 2.76**	(1) *t* = 4.254, *P* < 0.0001; (2) *t* = 2.401, *P* = 0.0214; (3) *t* = 3.484, *P* = 0.0013
	*w*Pip-	3.75 ± 4.33**	0.3 ± 0.57*	3.4 ± 4.02**	
12 h	*w*Pip+	7.95 ± 8.91**	0.7 ± 0.86	6.4 ± 7.05**	(1) *t* = 2.543, *P* = 0.0152; (2) *U* = 172.5, *P* = 0.4569; (3) *t* = 3.015, *P* = 0.0046
	*w*Pip-	2.3 ± 3.03**	0.7 ± 1.34	1.5 ± 1.76**	
24 h	*w*Pip+	6.25 ± 14.57**	2.05 ± 3.69**	1.1 ± 1.16**	(1) *U* = 80.5, *P* < 0.0012; (2) *U* = 110, *P* = 0.0114; (3) *U* = 110, *P* = 0.0139
	*w*Pip-	0.75 ± 1.45**	0.4 ± 0.94**	0.3 ± 0.66**	

*Abbreviations*: *PVP* pre-vitellogenic period, *VP* vitellogenic period, *w*Pip+ infected, *w*Pip- uninfected; nd, no data, *T* student t test, *MW* Mann-Whitney U- test, *P* *P*-value

*P < 0.05

**P < 0.02

^a^Undifferentiated follicles, considered as secondary in the table

^b^(1) statistics related to mean number of apoptotic follicular cells per ovary; (2) statistics related to mean number of primary follicles that have at least 1 apoptotic event; (3) statistics related to mean number of secondary follicles that have at least 1 apoptotic event

^c^All values for degrees of freedom for Student’s *t* - test: *df* = 38

Due to the impossibility of distinguishing follicles in the first and second day of the PVP, only the number of apoptotic follicular cells per ovary was counted on the first day, whereas on the second day the apoptotic events were all counted as they belonged to secondary follicles (Table 1, Additional file 4: Figure S4; Additional file 5: Table S1).

### Apoptosis of nurse/oocyte cells

**Fig. 2:**
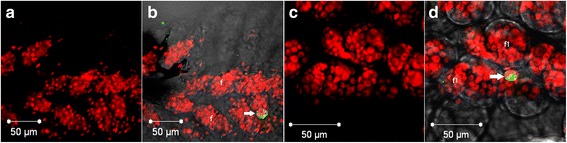
Photomicrographs of infected *Cx. quinquefasciatus* ovary under confocal microscopy on the second day of PVP (**a**, **b**) and on the third day of PVP (**c**, **d**). Only nuclei (**a**, **c**) and merge (**b**, **d**). Arrow in **b** and **d** indicates nuclei of apoptotic oocyte or nurse cell. *Key*: *red*, nuclei; *green*, apoptotic cell; *white*, visible light. *Abbreviations*: f, follicle; f1, primary follicle

Differently, from the follicular cells, very few oocytes or nurse cells were labelled with TUNEL. Labelling was seen only in infected individuals and at second (*n* = 7) and third day of the PVP (*n* = 1) (Fig. 2).

### Atresia

**Fig. 3:**
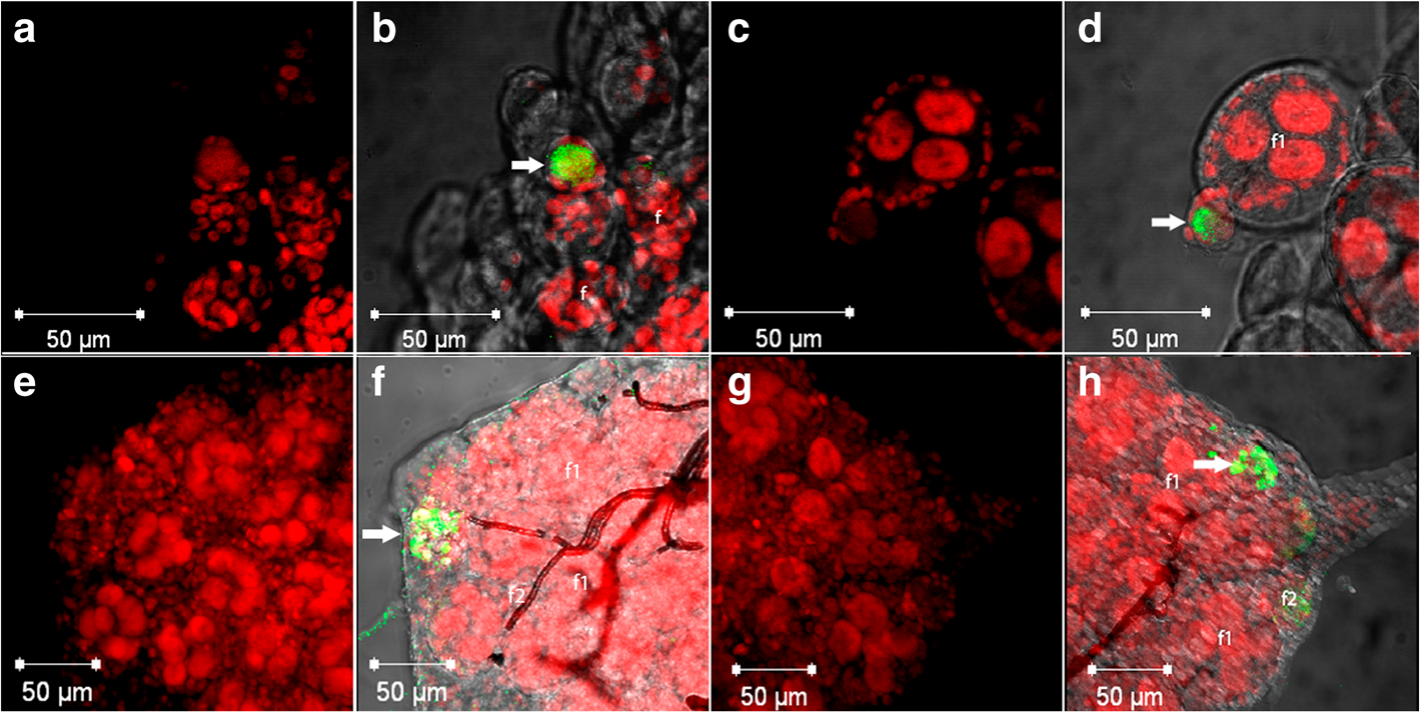
Photomicrographs of *Cx. quinquefasciatus* ovary under confocal microscopy on: **a**, **b** the second day of PVP of wPip+ ovary; **c**, **d** the fourth day of PVP of wPip+ ovary; **e**, **f** the fifth day of PVP of wPip+ ovary; **g**, **h** 12 h of VP of wPip- ovary. Only nuclei (**a**, **c**, **e**, **g**) and merge (**b**, **d**, **f**, **h**). Arrows indicate follicular atresia. *Key*: *red*, nuclei; *green*, atresia; *white*, visible light. *Abbreviations*: f, follicle; f1, primary follicle; f2, secondary follicle

When analysing microscope images, we considered follicular atresia when the whole follicle was labelled by TUNEL. Few atretic follicles were found at the observed times: second day (*n* = 3; Fig. 3a); fourth day (*n* = 3; Fig. 3b); and fifth day of the PVP (*n* = 1; Fig. 3c), and 12 h of the VP (*n* = 1; Fig. 3d). The atresia on the second and third day of the PVP apparently occurred in secondary follicles and on the fifth day of the PVP and 12 h of VP they were observed in primary follicles. These observed atresia events on PVP times frequently happen in wPip+ mosquitoes, and the only one observed in VP happened in wPip- mosquito.

## Discussion

During the five days of PVP, most apoptoses of follicular cells occurred earlier (days 1–3). This observation is compatible with the fact that this tissue was under proliferation and differentiation, as previously reported by other researchers (see [37]). Coherently, we also observed the lower occurrence of apoptosis on the last days of PVP (days 4 and 5) when the ovary was reaching the resting period.

Comparing the number of apoptotic cells in *w*Pip+ and *w*Pip-, we observed that this phenomenon occurs mostly in ovaries of infected females, especially in the second and third day of the PVP, and at 6 h, 12 h and 24 h of the VP. These apoptotic cells were observed mainly in the secondary follicles, which would become eggs in the second gonotrophic cycle. Coincidentally the second gonotrophic cycle (corresponding to secondary follicles) is when infected females laid fewer eggs [35], what lead us to pose a new hypothesis: the eggs reduction is caused by *Wolbachia*-induced apoptosis. A next project should then investigate this neo-hypothesis.

Although there were significantly more apoptotic cells in the primary follicles on the third and fifth day of PVP in *w*Pip- mosquitoes, total apoptoses in the ovary over time was low (< 2), and, considering our hypothesis, does not imply a significant change in the total number of eggs laid (approximately 200 eggs in the first gonotrophic cycle) as reported by our group [35].

It seems that apoptosis of follicular cells would not lead, in most cases, the follicle to death, because many follicles eventually present apoptosis, but only a few undergo atresia. A possible explanation for the follicle survival is the tissue repair systems, that have been described in response to apoptosis in *Anopheles* gut, via actin cone zipper [38], and via gut regeneration in the same mosquito species used in this work [39] and another possibility is that the microtubules of adjacent cells, responsible for the patency (intercellular channels) formation, can be involved in this epithelial restructuration. On the other hand, the apoptosis of few follicular cells could increase patency formation and, consequently, increase the absorption of yolk by the oocyte through the follicular cells, which would be beneficial for oocyte maturation. This possibility could result in the faster eggs maturation, previously observed in infected females [35].

Other studies concerning the occurrence of apoptosis in the germinal tissue to the absence of *Wolbachia* were performed in *Brugia malayi* [40], *Drosophila mauritiana* [41], and *Asobara tabita* [42], but in those cases, the *Wolbachia* relationship with the hosts are considered mutualistic, unlike that of *Culex*. However, the symbiotic relationship was considered parasitic with the induction of apoptosis in the germ cells of *Drosophila melanogaster* infected with a virulent strain (*w*MelPop) of *Wolbachia* [43]. The same strain of the bacteria was transfected into *Aedes albopictus*, which resulted in fewer eggs laid and follicular atresia was a possible cause of the reduction [44].

Pan et al. [19] found that transfection of *w*MelPop for *Aedes aegypti* caused an increase in transcription of genes related to the immune system of the mosquito, and unregulated transcription of antioxidant genes and ferritin gene, a protein responsible for storage of free iron. It is known that these factors are directly related to apoptosis induction [45]. In our view, the induction of apoptosis described in this work could, at least in part, be related to any of the results obtained by Pan et al. [19] being that the presence of *Wolbachia* would cause oxidative stress in the ovary cells.

In this work, we expected to find whole follicles in apoptosis (follicular atresia), but it was rarely observed using our method. In *Ae. aegypti*, atresia was detected between 26 h and 30 h after the blood meal [3], and in *Culex pipiens pallens* it occurred between two and three days after the blood meal [22, 23]. Our observations of ovaries during the vitellogenic period showed that at 12 h of VP, atresia occurred in a single ovarian follicle, and according to the descriptions noted above, atresia usually occurs later. Unfortunately, the formation of the chorion prevented the observation of follicles after 24 h in the VP, and we could not observe this phenomenon with the method utilised in this study. It is also notable that the TUNEL reagent is commonly used for cells and not for tissues. Therefore, TUNEL reagent may not have been an efficient method to detect apoptosis in tissues or huge cells because of its low penetration.

According to Clements [24], the occurrence of atresia begins during the resting period (days 4–5 of PVP), but in our study, we found six atretic ovarian follicles on days 2–4 of PVP. This is the first study to document this in mosquitoes. Chao & Nagoshi [46] and Timmons et al. [15] concluded that follicular cells in apoptosis might induce apoptosis in nurse cells or oocytes, leading to follicular atresia. In addition to the apoptosis in follicular cells, we observed eight nurse cells (or oocytes) in apoptosis and eight follicles in atresia in 320 ovaries analysed in this work, which although small, confirmed that this type of cell death occurs in *Culex quinquefasciatus* ovaries.

We cannot rule out that there are ten types of programmed cell death as described in *Drosophila*, which occur on different pathways than that of apoptosis [15], and our detection method was not sufficient to observe all of them. In addition, the confocal method was partly limited by the formation of the chorion, which prevented the passage of the laser. Unfortunately, the occurrence of some apoptoses and atresia seems to occur in this period [21, 24, 33, 47].

In this study, we firstly detected the occurrence of atresia during PVP. In addition, we observed follicular cells in apoptosis in the previtellogenic and early vitellogenic periods, but this phenomenon was rare in nurse and oocyte cells. Although apoptosis of follicular cells is a common occurrence in many organisms, it was not described in mosquitoes hitherto, and this work documents quantitatively and temporally its occurrence. Other approaches for future investigation on how *Wolbachia* influences reproduction of mosquitoes would be labelling *Wolbachia* for determine its location on mosquito tissues, and searching for apoptosis indicator genes/molecules acting differently between infected and uninfected mosquitoes.

## Conclusions

To the best of our knowledge, the follicular atresia is first reported in the mosquito previtellogenic period of *Cx. quinquefasciatus*. The occurrence of apoptosis in ovarian follicular cells is more common than we believed, and is increased in the presence of *Wolbachia*. Because more apoptosis correlates with fewer eggs in the second gonotrophic cycle, it is worth investigating the causal relationship between apoptosis occurrence and egg diminishing in *Wolbachia*-infected mosquitoes. The findings of this work may have implications for mosquito control and, principally, in the use of *Wolbachia* as a mosquito and pathogens control strategy.

## Additional files

Additional file 1: Figure S1. Agarose gels showing the successful removal of *Wolbachia* from all mosquitoes analysed following the generation F4 (**a**) and F5 (**b**) after antibiotics treatments. **a** Lanes 1–17: DNA of one F4 female per pool after tetracycline treatment. **b** Lanes 1–12: DNA of one F5 female per pool after tetracycline treatment. *Abbreviations*: Mw, molecular weight of 100 base pair; +, positive control (DNA of infected mosquito); −, negative control (without DNA) (TIFF 56155 kb)

Additional file 2: Figure S2. Photomicrographs of ovary development of infected *Cx. quinquefasciatus* under confocal microscopy. **a** First day of PVP. **b** Second day of PVP. *Key*: *red*, nuclei; *green*, apoptotic cell; *white*, visible light. *Abbreviations*: f, follicle; c, calyx (TIFF 1319 kb)

Additional file 3: Figure S3. Photomicrographs of ovary development of infected *Cx. quinquefasciatus* under confocal microscopy. (**a**, **b**) third day of PVP; (**c**, **d)** fifth day of PVP; (**e**, **f)** 24 h of VP. (**a**, **c**, **e**), only nuclei; (**b**, **d**, **f**), merge. Arrows indicate nuclei of oocyte or nurse cell; asterisks, nuclei of follicular cells. *Key: red*, nuclei; *green*, apoptotic cell; *white*, visible light. *Abbreviations*: f1, primary follicle; f2, secondary follicle; o, oocyte; nc, nuclei of nurse cell (TIFF 2175 kb)

Additional file 4: Figure S4. Photomicrographs of ovary development and chorion formation of *Cx. quinquefasciatus* under confocal microscopy. **(a**, **b)** ovary from wPip+, 36 h of VP; (**c**, **d**) ovary from wPip-, 48 h of VP; (**e**, **f**) ovary from wPip-, 72 h of VP. (**a)**, only nuclei; (**c**, **e**), only visible light; (**b**, **d**, **f**), merge. *Abbreviations*: f1, primary follicle; f2, secondary follicle; o, oocyte; nc, nuclei of nurse cell; c, chorion. *Key*: *red*, nuclei; *green*, apoptotic cell; *white*, visible light (TIFF 2169 kb)

Additional file 5: Table S1. Descriptive statistics of apoptosis occurrence of follicular cells from infected and uninfected *Cx. quinquefasciatus. Abbreviations*: PVP, previtellogenic period; VP, vitellogenic period; wPip+, infected mosquitoes; wPip-, uninfected mosquitoes; nd, no data; Max, maximum quantity per ovary; Min, minimum quantity per ovary; ^a^, number of follicles with at least one follicular apoptotic cell; ^b^, undifferentiated follicle considered as secondary (DOCX 15 kb)

Additional file 6: Figure S5. **a** Mean of apoptotic follicular cells per ovary. **b** Mean of primary follicles with at least one apoptotic follicular cell per ovary. **c** Mean of secondary follicles with at least one apoptotic follicular cell per ovary. *Abbreviations*: PVP, previtellogenic period; VP, vitellogenic period; wPip+, infected mosquitoes; wPip-, uninfected mosquitos; *bar*, standard deviation; ^a^, undifferentiated follicle considered as secondary follicle in the graphs (DOCX 20 kb)

## Abbreviations

PCD: programmed cell death
PVP: previtellogenic period
VP: vitellogenic period
*w*Pip-: *Wolbachia* uninfected group
*w*Pip+: *Wolbachia* infected group
