# Supplementary figures and images for: Effects of Wolbachia on ovarian apoptosis in Culex quinquefasciatus (Say, 1823) during the previtellogenic and vitellogenic periods
Source: Parasit Vectors. 2017 Aug 25;10:398. doi: 10.1186/s13071-017-2332-0 (PMC5574119; doi:10.1186/s13071-017-2332-0)

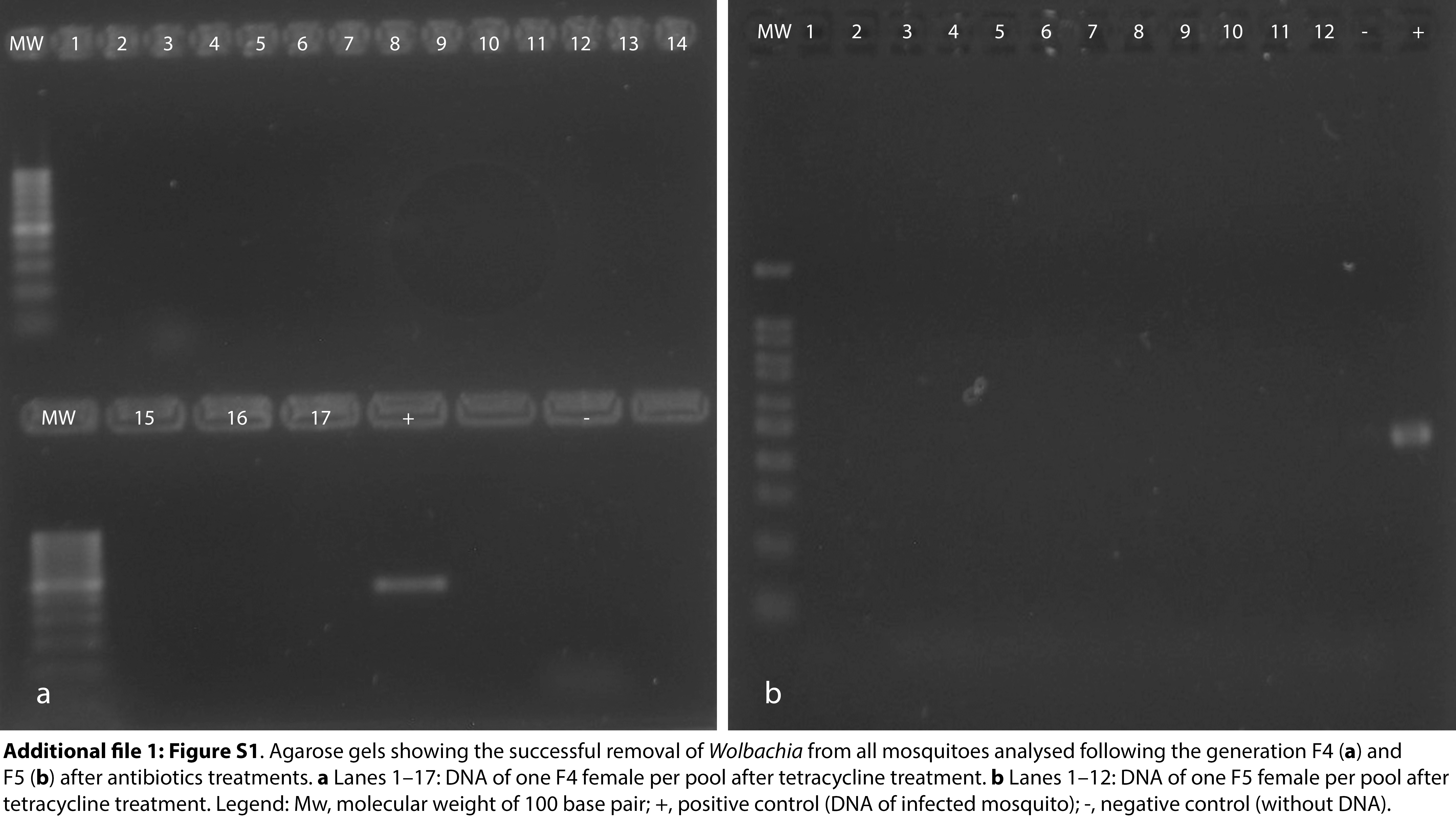

Supplement: Supplementary file 1 — Agarose gels showing the successful removal of Wolbachia from all mosquitoes analysed following the generation F4 (a) and F5 (b) after antibiotics treatments. a Lanes 1–17: DNA of one F4 female per pool after tetracycline treatment. b Lanes 1–12: DNA of one F5 female per pool after tetracycline treatment. Abbreviations: Mw, molecular weight of 100 base pair; +, positive control (DNA of infected mosquito); −, negative control (without DNA) (TIFF 56155 kb) [file 13071_2017_2332_MOESM1_ESM.tif]

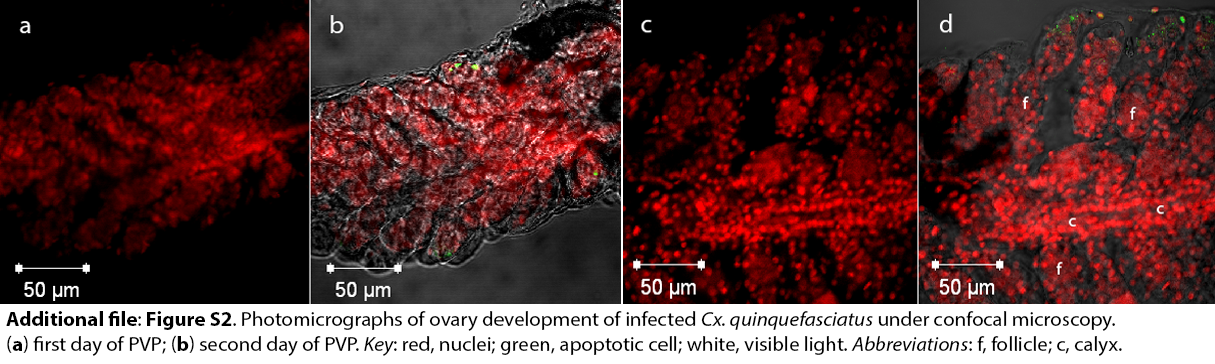

Supplement: Supplementary file 2 — Photomicrographs of ovary development of infected Cx. quinquefasciatus under confocal microscopy. a First day of PVP. b Second day of PVP. Key: red, nuclei; green, apoptotic cell; white, visible light. Abbreviations: f, follicle; c, calyx (TIFF 1319 kb) [file 13071_2017_2332_MOESM2_ESM.tif]

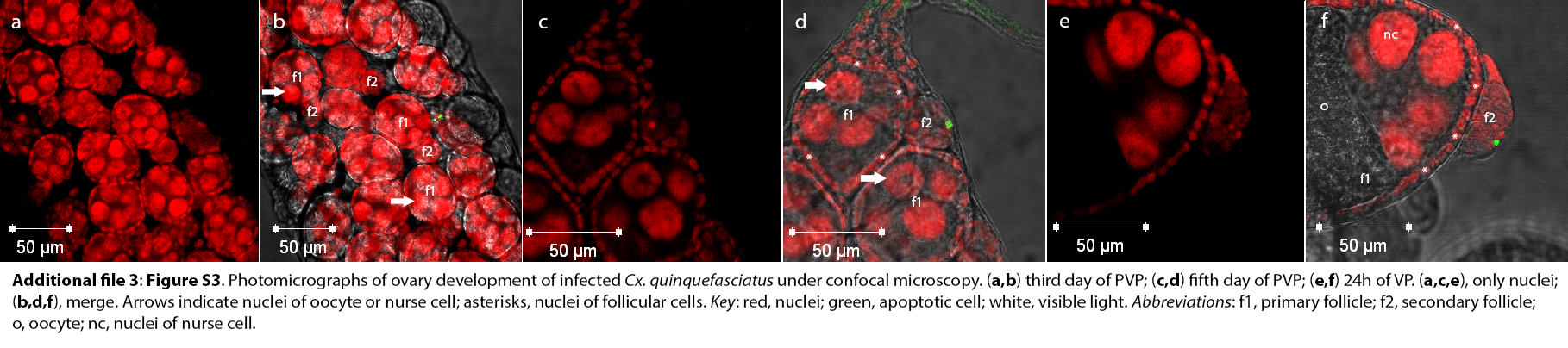

Supplement: Supplementary file 3 — Photomicrographs of ovary development of infected Cx. quinquefasciatus under confocal microscopy. (a, b) third day of PVP; (c, d) fifth day of PVP; (e, f) 24 h of VP. (a, c, e), only nuclei; (b, d, f), merge. Arrows indicate nuclei of oocyte or nurse cell; asterisks, nuclei of follicular cells. Key: red, nuclei; green, apoptotic cell; white, visible light. Abbreviations: f1, primary follicle; f2, secondary follicle; o, oocyte; nc, nuclei of nurse cell (TIFF 2175 kb) [file 13071_2017_2332_MOESM3_ESM.tif]

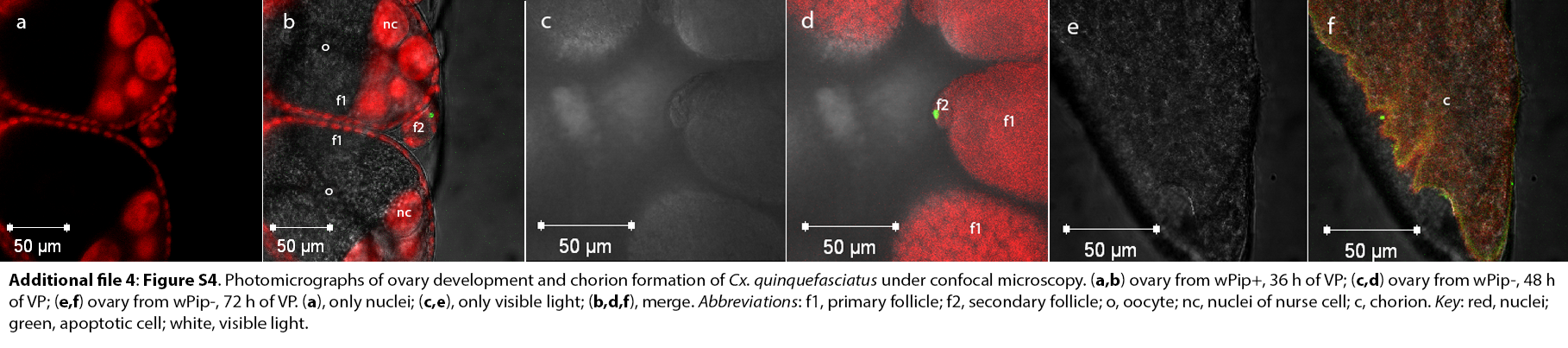

Supplement: Supplementary file 4 — Photomicrographs of ovary development and chorion formation of Cx. quinquefasciatus under confocal microscopy. (a, b) ovary from wPip+, 36 h of VP; (c, d) ovary from wPip-, 48 h of VP; (e, f) ovary from wPip-, 72 h of VP. (a), only nuclei; (c, e), only visible light; (b, d, f), merge. Abbreviations: f1, primary follicle; f2, secondary follicle; o, oocyte; nc, nuclei of nurse cell; c, chorion. Key: red, nuclei; green, apoptotic cell; white, visible light (TIFF 2169 kb) [file 13071_2017_2332_MOESM4_ESM.tif]
