# Supplementary material for: Effects of Wolbachia on ovarian apoptosis in Culex quinquefasciatus (Say, 1823) during the previtellogenic and vitellogenic periods
Source: Parasit Vectors. 2017 Aug 25;10:398. doi: 10.1186/s13071-017-2332-0 (PMC5574119; doi:10.1186/s13071-017-2332-0)
Supplement: Supplementary file 5 — Descriptive statistics of apoptosis occurrence of follicular cells from infected and uninfected Cx. quinquefasciatus. Abbreviations: PVP, previtellogenic period; VP, vitellogenic period; wPip+, infected mosquitoes; wPip-, uninfected mosquitoes; nd, no data; Max, maximum quantity per ovary; Min, minimum quantity per ovary; a, number of follicles with at least one follicular apoptotic cell; b, undifferentiated follicle considered as secondary (DOCX 15 kb) [file 13071_2017_2332_MOESM5_ESM.docx]

|  | **Apoptotic follicular cells per ovary** | | | **Primary follicle with apoptotic cell^a^** | | | **Secondary follicle with apoptotic cell^a^** | | |
| --- | --- | --- | --- | --- | --- | --- | --- | --- | --- |
|  | Max | Min | Median | Max | Min | Median | Max | Min | Median |
| **wPip+** |  |  |  |  |  |  |  |  |  |
| 1^st^ day (PVP) | 11 | 0 | 2 | nd | nd | nd | nd | nd | nd |
| 2^nd^ day (PVP) | 68 | 2 | 11.5 | nd | nd | nd | 22^b^ | 2^b^ | 10.5^b^ |
| 3^rd^ day (PVP) | 21 | 1 | 9 | 3 | 0 | 0 | 14 | 1 | 8 |
| 4^th^ day (PVP) | 13 | 0 | 3 | 1 | 0 | 0 | 12 | 0 | 3 |
| 5^th^ day (PVP) | 8 | 0 | 1 | 3 | 0 | 0 | 5 | 0 | 1 |
| 6h (VP) | 16 | 3 | 7.5 | 5 | 0 | 1 | 13 | 3 | 6.5 |
| 12h (VP) | 38 | 0 | 7.5 | 3 | 0 | 0.5 | 30 | 0 | 6 |
| 24h (VP) | 66 | 0 | 2 | 16 | 0 | 1 | 3 | 0 | 1 |
| **Sum** | 241 | 6 | 43.5 | 31 | 0 | 2.5 | 99 | 4 | 36 |
|  |  |  |  |  |  |  |  |  |  |
| **wPip-** |  |  |  |  |  |  |  |  |  |
| 1^st^ day (PVP) | 13 | 0 | 1 | ND | ND | ND | ND | ND | ND |
| 2^nd^ day (PVP) | 12 | 0 | 3 | ND | ND | ND | 12^b^ | 0^b^ | 2.5^b^ |
| 3^rd^ day (PVP) | 17 | 1 | 7.5 | 6 | 0 | 1 | 15 | 1 | 5.5 |
| 4^th^ day (PVP) | 9 | 0 | 2 | 1 | 0 | 0 | 8 | 0 | 1 |
| 5^th^ day (PVP) | 8 | 0 | 3.5 | 5 | 0 | 0.5 | 8 | 0 | 2 |
| 6h (VP) | 14 | 0 | 2 | 2 | 0 | 0 | 13 | 0 | 1 |
| 12h (VP) | 13 | 0 | 2 | 5 | 0 | 0 | 6 | 0 | 1 |
| 24h (VP) | 6 | 0 | 0 | 4 | 0 | 0 | 2 | 0 | 0 |
| **Sum** | 92 | 1 | 21 | 23 | 0 | 1.5 | 64 | 1 | 13 |

**Additional file 5. Table S1.** Descriptive statistics of apoptosis occurrence of follicular cells from infected and uninfected *Cx. quinquefasciatus.* *Abbreviations*: PVP, previtellogenic period; VP, vitellogenic period; wPip+, infected mosquitoes; wPip-, uninfected mosquitoes; nd, no data; Max, maximum quantity per ovary; Min, minimum quantity per ovary; ^a^, number of follicle with at least one follicular apoptotic cell; ^b^, undifferentiated follicle considered as secondary.
