# Supplementary material for: Effects of Wolbachia on ovarian apoptosis in Culex quinquefasciatus (Say, 1823) during the previtellogenic and vitellogenic periods
Source: Parasit Vectors. 2017 Aug 25;10:398. doi: 10.1186/s13071-017-2332-0 (PMC5574119; doi:10.1186/s13071-017-2332-0)
Supplement: Supplementary file 6 — a Mean of apoptotic follicular cells per ovary. b Mean of primary follicles with at least one apoptotic follicular cell per ovary. c Mean of secondary follicles with at least one apoptotic follicular cell per ovary. Abbreviations: PVP, previtellogenic period; VP, vitellogenic period; wPip+, infected mosquitoes; wPip-, uninfected mosquitos; bar, standard deviation; a, undifferentiated follicle considered as secondary follicle in the graphs (DOCX 20 kb) [file 13071_2017_2332_MOESM6_ESM.docx]

**a**

**b**

**c**

**Additional file 6.** **Figure S5.** **a** Mean of apoptotic follicular cells per ovary; **b** Mean of primary follicles with at least one apoptotic follicular cell per ovary; **c** Mean of secondary follicles with at least one apoptotic follicular cell per ovary. *Abbreviations*: PVP, previtellogenic period; VP, vitellogenic period; wPip+, infected mosquitoes; wPip-, uninfected mosquitos; *bar*, standard deviation; ^a^, undifferentiated follicle considered as secondary follicle in the graphs.
